# Virtuelle IT-Studienprojekte – Ein Erfahrungsbericht und Handlungsempfehlungen für Betreuende

**DOI:** 10.1365/s40702-021-00790-4

**Published:** 2021-09-29

**Authors:** Julien Hofer, Ralf Knackstedt

**Affiliations:** Abteilung Informationssysteme und Unternehmensmodellierung, Institut für Betriebswirtschaft und Wirtschaftsinformatik, Hildesheim, Deutschland

**Keywords:** Virtualisierung, IT-Studienprojekt, Agiles Vorgehen, Erfahrungsbericht, Virtualization, Capstone Project, Agile Approach, Experience Report

## Abstract

Der vorliegende Beitrag berichtet über die Erfahrungen eines virtuellen Studienprojekts, welches an einer deutschen Universität durchgeführt wurde. Dabei wird ein allgemeiner Überblick über die Virtualisierung von Lernen und Lehren gegeben und ein Fokus auf die agile und virtuelle Herangehensweise gesetzt. Es wird auf wichtige Aspekte der Kollaboration in virtuellen Settings eingegangen, das Setting bei einem IT-Studienprojekt allgemein beschrieben und dann ausführlich über ein konkretes, virtuelle, agiles IT-Studienprojekt in einem IT-Studiengang berichtet. Dieses Projekt befasste sich mit der Konzeption und Gestaltung eines sprachbasierten Conversational User Interfaces im Kontext von Design Thinking. Aufgrund der COVID-19-Pandemie wurde das einjährige Projekt komplett virtuell durchgeführt. Dozierender und Studierende haben also vollkommen digital mit Werkzeugunterstützung gelehrt, gelernt und gearbeitet. Mit diesem Beitrag liefern wir Erfahrungen und Anhaltspunkte, die als Basis für die Gestaltung und Durchführung anderer virtueller und agiler IT-Studienprojekte verwendet werden können. Wir ergänzen den Erfahrungsbericht schließlich mit konkreten Handlungsempfehlungen zu verschiedenen Gestaltungs- und Durchführungsaspekten, die Interessierten – Lehrenden an Hochschulen sowie Praktizierende aus Wirtschaft und Verwaltung – als Hilfestellungen dienen können.

## Abstract (en)

The following contribution is a report on a virtual IT capstone project which has been undertaken at a German university. We provide a general overview on virtual learning and teaching. Thereby, we explain selected theoretical underpinnings of agile and virtual methods in the context of universities. We provide knowledge on virtual collaboration drawing upon selected relevant literature. Then, we describe IT capstone projects in general, before we exhaustively report from our experience with a specific virtual, agile IT capstone project. The latter aimed at developing a Conversational User Interface in the realm of Design Thinking. Due to the Covid-19-pandemic the entire project has been executed fully virtual over a period of one year. Hence, supervisor’s teaching and learning on the part of students has been done completely digital supported by IT tools. With this practical-oriented article, we provide experience and design parameters serving as a basis for the design, development and execution of future virtual and agile IT capstone projects. We complement our report with recommendations on various design- and execution-related aspect in order to support not only academic supervisors, but also interested practitioners from areas such as business and public administration.

## Einleitung und Motivation

Der Digitale Wandel wirkt sich auf nahezu alle Bereiche unseres Lebens aus. Der Bildungsbereich an Hochschulen ist davon ebenso betroffen wie die Wirtschaft (vgl. Robra-Bissantz und Siemon 2018). Es ergeben sich dabei aber auch Potenziale zwischen Hochschulen und Wirtschaft, insbesondere Formate, die Digitalkompetenz auf beiden Seiten stärken (vgl. Sinell 2020). Vor diesem Hintergrund ändert sich auch die Disziplin der Wirtschaftsinformatik, insbesondere die Art und Weise wie gelehrt wird und wie Studierende lernen. Hier treten im Zuge der Digitalisierung Veränderungen auf. Besonders im Jahr 2020 mit seinen Ausgangsbeschränkungen aufgrund der Covid-19-Pandemie waren Präsenzformate oftmals nicht möglich, sodass Dozierende bestehende Lehrformate anpassen oder neue Formate entwickeln mussten.

Ein wichtiger Baustein im Wirtschaftsinformatik-Studium sind Projektarbeiten. Neben klassischen Lehrformaten bieten Projekte die Möglichkeit über einen längeren Zeitraum an einer Problemstellung selbstständig als Gruppe zu arbeiten. Beispielhafte Problemstellungen und Teilaufgaben sind u. a. Konzeption und Entwicklung einer Datenbank, Entwurf einer Software-Architektur oder Literaturanalysen. Studierende bauen so Kompetenzen in den Bereichen Projektmanagement, Problemlösung, wissenschaftliches Arbeiten, Software-Entwicklung und Kollaboration auf. Im Rahmen dieser Projektarbeiten wird oft der akademische Bereich mit Unternehmen und Organisationen außerhalb der Hochschule verknüpft. So können Studierende schon während des Studiums praxisorientiert lernen und Fähigkeiten erwerben, die sie künftig in ihrem Beruf einsetzen können.

Die Durchführung von IT-Studienprojekten an unserer Universität stand vor großen Herausforderungen, die durch die Covid-19 Pandemie und die damit verbundenen Eindämmungsmaßnahmen, u. a. Ausgangsbeschränkungen und Kontaktverbote, hervorgerufen wurden. Sämtliche organisatorische Prozesse mussten von den Betreuenden virtuell abgebildet werden, d. h. Information über mögliche Projekte, Bewerbung für Projekte, Projekt-Auftaktveranstaltung, Team-Building und ähnliches. Aufgrund fehlender Erfahrungswerte war es nicht leicht den Rahmen zu gestalten, wie z. B. den Grad der Strukturierung, die Kommunikationskanäle, die Technologien und Medien, sowie der Umgang mit unvorhersehbaren Ereignissen. Die größte Herausforderung für Studierende im eigentlichen Projekt bestand im verteilten Arbeiten. Die Projektbeteiligten durften sich nie gemeinsam an einem Ort treffen, da dies untersagt war. Nur Arbeiten aus der Ferne, sog. remote work, war möglich. Erschwerend kam hinzu, dass synchrones Arbeiten nur mittels Kollaborationstechnologien möglich war. Der Anteil asynchronen Arbeitens war sehr hoch. Dementsprechend stieg der Kommunikations- und Koordinationsaufwand an. Das virtuelle, verteilte Arbeiten wurde komplexer. Im konkreten IT-Studienprojekt, über das hier berichtet wird, kannten sich zudem die meisten Teilnehmenden nicht vorab. Das gegenseitige Kennenlernen musste ebenso rein virtuell erfolgen. Außerdem war das Stresslevel durch Parallelveranstaltungen, Tätigkeiten neben dem Studium, sowie Lebens- und Wohnsituation grundsätzlich hoch.

Gleichzeitig wächst der Bedarf an Fach- und Führungskräften in der Wirtschaft, die kompetent im Umgang mit virtuellem Arbeiten und Kollaborationstechnologien sind, sowie mit den Herausforderungen dieser Arbeitsform vertraut sind, insbesondere in Bezug auf Kommunikation (vgl. Goldberg und Howe 2015, S. 1773). In Zukunft wird es für Arbeitnehmende immer wichtiger im Team arbeiten zu können und ihre Kooperationskompetenz stets weiterzuentwickeln, sodass sie innerhalb ihrer Organisation, sowie organisationsübergreifend erfolgreich netzwerken können (vgl. von der Oelsnitz 2018; Robra-Bissantz und Siemon 2018).

Vor diesem Hintergrund möchten wir unsere Erfahrungen mit virtuellen IT-Studienprojekten teilen. Wir möchten über ein konkretes IT-Studienprojekt berichten und dabei positive und negative Aspekte näher beleuchten. Wir stellen daher das Format des virtuellen, agilen IT-Studienprojekts vor und berichten über die konkrete Projektdurchführung im Sommersemester 2020 und Wintersemester 2021, bei dem 9 Studierende der Wirtschaftsinformatik ein Conversational User Interface im Kontext von Design Thinking entwickelt haben.

Dazu werden zunächst im zweiten Kapitel zentrale Konzepte und verwandte Arbeiten vorgestellt und erläutert. Anschließend erklären wir die allgemeine Struktur von IT-Studienprojekten an unserer Universität, bevor wir im vierten Kapitel über die Struktur des konkreten IT-Studienprojekts aus dem Sommersemester 2020/Wintersemester 2021 berichten, welches den zentralen Gegenstand dieses Beitrags bildet. Wir schildern hierbei Erfahrungen aus Betreuendenperspektive und ergänzen diese mit Erkenntnissen aus Interviews mit vier Studierenden und der Projektdokumentation. Im vorletzten Kapitel leiten wir auf Basis der gesammelten Erfahrungen Handlungsempfehlungen für Betreuende ab. Im sechsten und letzten Kapitel beenden wir den Beitrag mit einer Zusammenfassung und einem Ausblick, der ausgewählte Gestaltungsaspekte für zukünftige IT-Studienprojekte beinhaltet.

## Zukunftsorientiertes Lernen und Lehren: Virtuell & Agil

Zunächst wollen wir ein grundlegendes Verständnis der zentralen Konzepte und verwandten Arbeiten schaffen, die den Rahmen für diesen Beitrag bilden.

Charakteristisch für jede Art größerer Projekte ist *Kollaboration*. Kollaboration beschreibt das Bestreben einer Gruppe von Menschen ein gemeinsames Ziel zu erreichen (Benke und Maedche 2018). Kollaboration besteht aus drei Teilbereichen: Kommunikation, Koordination, Kooperation (ebd.). Dabei interagieren verschiedene Personen miteinander, um bedeutungstragende Inhalte zu übermitteln (ebd). Koordination beschreibt Kommunikationsprozesse zur Abstimmung über Projektaufgaben (ebd.). Kooperation findet statt, wenn sich mehrere Individuen der Gruppe oder die gesamte Gruppe auf ein Ziel verständigt und gemeinsam auf dessen Erreichung hinarbeiten (ebd.).

*Virtuelle Kollaboration *bezeichnet eine Form der Zusammenarbeit zwischen Menschen, die sich an unterschiedlichen Orten befinden, nicht immer zur gleichen Zeit miteinander arbeiten und zur Unterstützung von Aktivitäten der Kommunikation, Kooperation und Koordination auf Informations- und Kommunikationstechnologie zurückgreifen (Benke und Maedche 2018). Als virtuelle Teams werden *„Gruppen von Mitarbeitern verstanden, die geografisch und zeitlich verteilt sind und mittels Technologie zusammengeführt werden, um eine organisatorische Aufgabe zu erfüllen.“ *(Benke und Maedche 2018) Neben dem Aspekt der geographischen Verteiltheit ist virtuelle Kollaboration durch virtuelle Kommunikationsmethoden gekennzeichnet. (Goldberg und Howe 2015). Um Kollaborationstechnologien handelt es sich dann, wenn mehrere Benutzende mittels einer Technologie zusammenarbeiten und sich gegenseitig dabei sehen können, bspw. MIRO^1^.

Kooperation spielt auch eine wesentliche Rolle bei* IT-Studienprojekten.* Diese zeichnen sich durch eine Reihe von Aspekten aus. Ziel ist es ein umfangreiches Artefakt zu entwickeln, bspw. ein Informationssystem oder eine Komponente eines komplexen Informationssystems und dabei im Studium erworbendes Wissen praktisch anzuwenden und auf ein konkretes Problem zu übertragen (Majanoja und Vasankari 2018). Es dauert in der Regel 6 Monate im Bachelor und 2 Semester im Master, also ein Jahr (Mahnic 2012). Dementsprechend ist die Projektaufgabe im Vergleich zu einer Gruppenarbeit komplexer, d. h. die Studierenden müssen eine wesentlich umfangreichere Problemstellung bearbeiten. Diese muss zunächst analysiert und verstanden werden, bevor sie in bearbeitbare Teilprobleme zerlegt werden kann. Aufgaben, Rollen und Verantwortlichkeiten müssen verteilt werden. Methoden des Projektmanagements müssen angewendet werden, u. a. Planung, Kontrolle, Steuerung. Die Gruppe muss sich organisieren, d. h. Strukturen zur Kommunikation, Koordination und Kooperation aufbauen. Zudem müssen die Studierenden Dokumentationsziele erreichen, d. h. kontinuierliche Projektdokumentation, Zwischen- und Abschlusspräsentation. Sie müssen theoretisch erworbenes Wissen praktisch anwenden und neue Lösungswege finden. Die Studierenden müssen als Gruppe agieren und durch Situationen der Unsicherheit navigieren. Das Lehrziel besteht darin, dass die Studierenden individuelle Kompetenzen, sowie gruppenbezogene Kompetenzen aufbauen, die sie später im Beruf einsetzen können.

*Agile IT-Studienprojekte* können als agil bezeichnet werden, wenn sie explizit Vorgehensweisen wie Scrum oder Extreme Programming nutzen und nicht dem Wasserfall-Paradigma folgen. Mit agilen Ansätzen können dabei positive Ergebnisse erzielt werden (Alshayeb et al. 2018). Studierende sind in agilen Settings gezwungen schneller und öfter mit der Kundenseite zu interagieren. Diese Flexibilität spiegelt sich auch in der Selbstorganisation des Teams wieder. So können in jedem Sprint neue Ziele fokussiert werden und das Team kann selbst die Ressourcenallokation steuern. Agile Vorgehensweisen im universitären Kontext bieten den Vorteil, dass Studierende schneller eine komplette Iteration von Anforderung, Entwurf, Entwicklung und Testen erleben und dadurch motivierter sind (Alshayeb et al. 2018).

Basierend auf diesem Verständnis definieren wir *virtuelle, agile IT-Studienprojekte* als solche, bei denen virtuelle Teams von Studierenden unter Nutzung von Kollaborationstechnologien über einen Zeitraum von mindestens 6 Monaten und unter Anwendung von Methoden, Techniken und Werkzeugen des agilen Arbeitens eine komplexe Projektaufgabe lösen, wobei sie unter Beweis stellen, dass sie im Studium erworbenes Wissens auf einen konkreten Problem- bzw. Anwendungsfall transferieren können.

## Format und Struktur des IT-Studienprojekts

In diesem Kapitel beschreiben wir das Konzept und den Aufbau des IT-Studienprojekts. Wir erläutern kurz jede Phase und gehen auf ausgewählte Kollaborationsaspekte ein.

An unserer Universität sind IT-Studienprojekte phasenorientiert strukturiert: Bewerbung, Bearbeitung, Präsentation. Dabei sollen die Studierenden ihre methodischen und sozialen Kompetenzen in der eigenverantwortlichen Projektarbeit entwickeln. Dabei werden u. a. Methoden des Projektmanagements, Leitung und Moderation von Gruppensitzungen und die Projektdokumentation adressiert.

In der *Bewerbungsphase* können sich Studierende auf verschiedene Projekte bewerben. Jedes Projekt wird in einem gesonderten Termin vorgestellt. In diesem Termin kann entweder ein Projekt oder mehrere Projekte verschiedener Betreuender vorgestellt werden. Für jedes Projekt wird der thematische Hintergrund und die zugrundeliegende Problemstellung erklärt. Anschließend können die Studierenden Fragen stellen. Basierend darauf beginnen die Studierenden mit ihren Bewerbungen für ihr präferiertes Projekt. Dies erfolgt in der Regel per E‑Mail oder über das Lern-Management-System der Universität. Oft bewerben sich Studierende direkt in der Kick-Off-Veranstaltung bei Dozierenden.

Dann erfolgt die *Bearbeitungsphase*, d. h. ein konkretes Projekt startet. In einer Auftaktveranstaltung, dem sog. Kick-Off, versammeln sich alle für das Projekt angemeldeten Studierenden und die Betreuungsperson. Dabei wird das Thema nochmals tiefer erläutert und konkrete Fragen der Studierenden beantwortet. Im Regelfall erfolgt dies in einem Seminar- bzw. Vorlesungsraum an einem bestimmten Termin. Im Rahmen dieses Termins einigen sich alle Projektbeteiligten auf einen festen, wiederkehrenden Termin an einem festen Ort, um kontinuierliche Projektbesprechungen durchzuführen. Die Terminfindung war bei vergangenen Projekten oft eine Herausforderung, wenn die Gruppenstärke relativ groß war. In den ersten vier Wochen des Projekts ist der Betreuungsaufwand eher hoch, da Unsicherheit bei den Studierenden zu Problemstellung, Vorgehensweise und Organisation herrscht. Die restliche Zeit dient der eigenverantwortlichen Bearbeitung durch das Projekt-Team.

Zum Ende des IT-Studienprojekts gibt es eine *Präsentationsphase.* Die Studierenden müssen dabei die Ergebnisse ihres Projekts vor allen Stakeholdern präsentieren. Die Präsentation kann dabei in Räumlichkeiten der Hochschule oder bei externen Organisationen erfolgen. Nach der Präsentation steht eine offene Diskussion an, bei der die Projektgruppe mit den Stakeholdern die Ergebnisse der Präsentation, sowie die Präsentationsleistung per se diskutiert. Engpässe bei Raumkapazitäten, sowie technische Hürden können hier Probleme verursachen. Die Studierenden müssen hier eigenverantwortlich vorsorgen.

Parallel zu Bearbeitungs- und Präsentationsphase erfolgt die *Dokumentationsphase*. Darin reflektieren und beschreiben die Studierenden die gesamt Projektdurchführung, u. a. Problemanalyse, Projektplan, Quellcode-Dokumentation etc. Erfahrungsgemäß wird qualitativ besser dokumentiert, wenn kontinuierlich und nicht endfällig dokumentiert wird.

Während des gesamten IT-Studienprojekts spielt *Kommunikation* eine entscheidende Rolle. Hier werden verschiedene *Kanäle* genutzt, u. a. E‑Mails, Nachrichten im Lern-Management-System, sowie persönliche Gespräche.

Eine Herausforderung für jede Projektgruppe besteht darin, eine für sich geeignete Balance zu finden, die dem Projektziel dienlich ist. Auch die Frequenz ist wichtig, d. h. wie viel Kommunikation pro Zeiteinheit. Bei Teammitgliedern, die sich zu Beginn nicht kennen muss außerdem eine adäquate *Art und Weise* der Kommunikation gefunden werden, bevor effektiv kommuniziert werden kann.

Weiterhin müssen Aufgaben beschrieben, verteilt und *koordiniert *werden. Es kommt bei der Organisation grundsätzlich auf das angestrebte Vorgehen innerhalb des IT-Studienprojekts an, d. h. inwiefern Studierende sich selbst organisieren oder ob Betreuende sehr viel vorstrukturieren, bspw. nach dem Wasserfall-Modell.

Eng verknüpft damit ist *Kooperation*, die oftmals eine Schwierigkeit darstellt für Studierende. Bei Gruppen mit Teilnehmenden, die keine oder wenig Projekterfahrung haben, entstehen häufig Schwierigkeiten durch Überforderung, da bspw. viele unbekannte Werkzeuge zur Nutzung vorgeschlagen werden oder weil erfahrenere Teilnehmende mehr entscheiden möchten.

Kollaboration, der Dreiklang aus Kommunikation, Koordination und Kooperation, stellt eine der zentralen Herausforderungen im IT-Studienprojekt dar, wenn mehrere heterogene Studierende als Gruppe ein gemeinsames Ziel über einen längeren Zeitraum erreichen sollen.

## Erfahrungen aus dem Studienprojekt „Gestaltung und Konversation eines Conversational User Interfaces“

Im folgenden Abschnitt berichten wir über die Erfahrungen aus einem konkreten IT-Studienprojekt mit Masterstudierenden. Dieses erstreckte sich über 2 Semester im Zeitraum von 2020 bis 2021, d. h. unter den besonderen Umweltbedingungen der Covid-19-Pandemie und den damit verbundenen Maßnahmen der Regierungen und Verwaltungen. Deshalb wurde dieses Projekt vollständig, von der Bewerbung des Projekts bis hin zur Präsentation der Endergebnisse, virtuell durchgeführt.

Die Aufgabenstellung des Projekts war die Gestaltung und Implementierung eines Conversational User Interfaces (CUI) zur Unterstützung eines Design Thinking Kurses. Das Projekt kann als *brownfield project* charakterisiert werden, da es auf bestehenden IT-Prototypen aufgebaut wurde. Es sollte ein sprachbasiertes CUI mit Hilfe eines Alexa Echos implementiert werden. Im Folgenden berichten wir, wie die konkreten Projektphasen virtuell durchlaufen wurden, welche Tools zum Einsatz kamen und welche besonderen Vorkommnisse es gab. Schließlich reflektieren wir positive und negative Aspekte der Durchführung.

In der *Bewerbungsphase *konnte keine Präsenzveranstaltung vor Ort stattfinden. Seitens der Lehrorganisation gab es die Vorgabe, dass eine Präsentation, eine textuelle Beschreibung oder Video bereitzustellen ist. Diese elektronische Datei sollte dann Studierenden im Lern-Management-System vor dem Start des Semesters zur Verfügung gestellt werden. Wir haben ein Video erstellt, in welchem der Dozierende gut sichtbar ist und über das Thema, mögliche Technologien und geeignete Organisationsformen referiert hat. Damit verbunden war ein hoher Aufnahme- und Bearbeitungsaufwand. Die eingesetzten Werkzeuge (Smartphone, Diktiergerät, iPad-Anwendung für Schnitt) sind für den Privatgebrauch geeignet, d. h. kein professionelles Aufnahme-Setting wurde genutzt. Die gesamte Produktion des Videos hat einen Arbeitstag gedauert aber es war uns wichtig, dass die Vorstellung des Projekts eine persönliche Note hat, um distanzmindernd zu wirken. Die Resonanz der Studierenden war positiv und 10 Studierende haben sich initial beworben, von denen letztlich 9 als Projektgruppe das IT-Studienprojekt gestartet haben.

Dann erfolgte die *Bearbeitungsphase.* Zu Beginn standen datenschutztechnische Herausforderungen bei der Entscheidung für eine Videokonferenzsoftware. Letztlich fiel die Entscheidung auf Zoom. Ein Grund dafür lag in der Erfahrung des Betreuenden mit dieser Software und der bereits vorhandenen Educator License. Im Vergleich zu bspw. Big Blue Button wurden weniger Lastprobleme erwartet. Zudem waren Studierende im Umgang bereits durch andere Lehrveranstaltungen geübt. Damit die Verwendung dieser Applikation datenschutzkonform geschehen konnte wurde von den Studierenden einerseits eine schriftliche Einverständniserklärung eingeholt, in der das Einverständnis zur Nutzung bestätigt und sich verpflichtet wurde keine audiovisuellen Mitschnitte über die Software zu erstellen. Weiterhin wurde darauf geachtet, dass das Hosting der Videokonferenzserver in Deutschland stattfindet.

In der ersten Sitzung des Projekts wurde in einer Videokonferenz ein lockeres Gespräch zum *Kennenlernen* geführt. Dabei sollten die Teilnehmenden sich vorstellen und erzählen wer sie sind, warum sie das Thema interessiert und welchen Hobbies sie nachgehen. Es wurde um Anschalten der Videokameras gebeten. Als Eisbrecher stellte sich der Betreuende vor, zeigte sein privates Arbeitszimmer und sogar seinen Hund. Dadurch wurde eine angenehme Atmosphäre geschaffen, die es den Teilnehmenden ermöglichte sich ungezwungen kennenzulernen. In dieser Projektgruppe kannte niemand mehr als 2 Personen vorab, d. h. es war größtenteils kein eingespieltes Team.

Der Betreuende hat die *Organisation* erläutert und einen Rahmen vorgestellt, der an das agile Vorgehen nach Scrum angelehnt war. Wir stellen dessen Hauptbausteine nachfolgend kurz vor:

- ***Daily***: Das Daily fand in einer besonderen Form statt. Da wir den Studierenden nicht auferlegen wollten sich zu einem bestimmten Zeitfenster für maximal 15 min in einer Telefon- oder Videokonferenz zu treffen wurde eine selbstentwickelte Webanwendung bereitgestellt, welche das Datum und die Antworten auf die Fragen „Was habe ich gemacht?“, „Was werde ich tun?“ und „Was gab es für Probleme?“ abfragt. Die Studierenden wurden gebeten täglich, unabhängig von der Uhrzeit, diese drei Fragen zu beantworten. Falls die Studierenden an einem Tag keine projektrelevanten Aufgaben bearbeitet haben, dann sollten sie dieses mit „nichts“ kennzeichnen.
- ***Weekly***: Zur Beratung wurde ein wöchentlicher Termin angesetzt bzw. vom Supervisor angeboten, welcher vor allem in der ersten Phase stark für die Unterstützung bei der technischen Einarbeitung genutzt wurde.
- ***Sprint Planning und Review***: In einem Turnus von zwei Wochen fand mit dem Supervisor (betreuender Dozent) ein Termin zur Berichterstattung und Planung der weiteren Schritte statt. Dabei berichtete einer der Studierenden über den im vorherigen Planning geschätzten Aufwand und den realen geleisteten Aufwand. Ebenfalls wurden in diesem Termin die Ergebnisse aus dem Sprint vorgestellt. Weiterhin wurde hier viel Platz gelassen für das Stellen von Fragen oder ggf. hat der Supervisor Hinweise gegeben wie ein bestimmtes Problem gelöst werden kann. Dabei wurde darauf geachtet, dass dies nicht als direkte Aufgabe vom Supervisor formuliert war sondern im Sinne eines Beratungsgesprächs Lösungsoptionen aufgezeigt wurden.
- ***Sprint***: Der Sprint erfolgte stets über 2 Wochen und wurde von den Studierenden eigenverantwortlich durchgeführt, d. h. der Supervisor nahm keinen Einfluss auf die Aufgabendefinition und -verteilung. Gleichzeitig stand der Supervisor für jegliche Frage in einem asynchronen Austausch per E‑Mail für Fragen zur Verfügung. Bei komplexeren Themen, die meist technischer Natur waren, wurden ad-hoc Videokonferenzen abgehalten.

Nach der Darstellung des Organisationsrahmens wurde vom Supervisor ein erster Überblick über Conversational User Interfaces im Allgemeinen und über sprachbasierte im speziellen gegeben. Dabei wurde auf Amazon Alexa und einige Vorarbeiten eingegangen. Weiterhin wurde über die technische Infrastruktur und die Zielvorstellung aus Betreuendensicht gesprochen. Der Betreuende erwähnte dabei mögliche Herangehensweisen, ohne dass eine verpflichtende Arbeitsform vorgegeben wurde.

In den darauf folgenden Sprint-Meetings wurde von einer Person aus der Gruppe die jeweils geschätzten Storypoints und die jeweils erreichten Storypoints berichtet. Der Supervisor fragte bei einer zu großen Abweichung nach, ob es Probleme beim jeweiligen Sprint gab. Wichtig war hierbei klar auszusprechen, dass diese Frage nicht „wertend“ sondern im Sinne der Verbesserung für die nächsten Sprints gemeint ist. Daraufhin wurde auch über Probleme wie z. B. beschränkter Datenbankzugriff oder aktuelle Prüfungen berichtet. Der Supervisor agierte als Coach, nicht als Instruktor oder hierarchischer Vorgesetzter.

In den ersten Weekly-Meetings wurde in erster Linie mit den Studierenden des Entwicklungsteams gearbeitet, das sich um die Entwicklung einer Anbindung an ein vorhandenes System und der Implementierung von Alexa-Skills innerhalb der Alexa-Developer-Console beschäftigt hat. Die Gruppe hat dieses Unter-Team selbst geformt. Da wir die Erfahrung gemacht haben, dass das Aufsetzen von Entwicklungsumgebungen auf verschiedenen Betriebssystemen immer wieder zu Schwierigkeiten führt, haben wir uns für die Verwendung einer Container-Technologie (Docker) entschieden und konnten eine vorgefertigte Entwicklungsinfrastruktur zur Verfügung stellen. In den weiteren Weekly-Meetings wurden entweder Fortschritte vorgestellt oder es wurde sich mit einem spezifischen Problem beschäftigt. Die Teilgruppe der Studierenden, welche sich mit dem Alexa-Skill und der dazugehörigen Abfragen von der Plattform beschäftigten, hatten keine Erfahrung in der genutzten Programmiersprache und des verwendeten Plattform-Systems. Gegen Ende des Projekts wurde dieser Termin auch kurzfristig für Klärungsbedarfe genutzt. Wir nehmen an, dass durch die kontinuierliche Unterstützung des Dev-Teams, die Hürde für die anderen Mitglieder diesen Termin zu nutzen gefallen ist.

Die *Präsentation* der Projektergebnisse lief über eine Videokonferenz. Dabei haben die Studierenden berichtet, dass sie sich mit dem Thema Conversational User Interface (CUI) bisher wenige Berührungspunkte im Studium hatten. Das IT-Studienprojekt konnte ihnen aber einen vertiefenden Einblick geben.

Weiterhin wurde von einem anfänglichen Problem berichtet, bei dem nicht genügend Daten für die Durchführung einer Aufgabe verfügbar waren. Es fehlten Instanzen für eine Design-Thinking-Methodendatenbank, wodurch das Entwicklungsziel gefährdet war. Deshalb entschied sich die Gruppe dafür, die Methodendatenbank zu verfeinern und durch eine verbesserte Struktur zu ersetzen. Dabei wurden weitere Methoden recherchiert, so dass die Methodendatenbank gewachsen ist. Um diese Methoden mit Alexa abfragen zu können wurden Intents entwickelt, konzipiert und per Screensharing mit Testnutzern getestet. Die Erkenntnisse sind dann in einem Vorgehensmodell für weitere CUI-Projekte verarbeitet worden.

## Handlungsempfehlungen

Auf Basis der Erfahrungen aus dem virtuellen IT-Studienprojekt haben wir die folgenden Handlungsempfehlungen abgeleitet.

### Agile Vorgehensweise

Die Unsicherheiten und plötzlich auftretenden Probleme konnten vom Projektteam weitgehend eigenständig gelöst werden. Die agile Vorgehensweise, die Flexibilität und das hohe Maß an Selbstorganisation erwiesen sich hier als erfolgreich. Wir nehmen an, dass dies bei einer vergleichsweise starren Vorgehensweise so nicht der Fall gewesen wäre. *Wir empfehlen für komplexe, neuartige Problemstellungen daher eine agile Vorgehensweise.*

### Nahbarkeit

Die Betreuungsperson trat weniger als Vorgesetzter auf, mehr als Coach. So wurde versucht nahbar zu wirken in einer Zeit, die sehr von Distanz geprägt war. *Wir empfehlen daher auf eine nahbare Art der Betreuungsperson zu setzen und keinen strikten Führungsstil.*

### Strukturierungsgrad

Aufgrund der hohen Unsicherheiten im Projekt, sowie im Privatleben der Studierenden und Betreuenden in der Covid-19-Pandemie wurde weniger vorstrukturiert als in bisherigen Projekten seitens der Betreuungsperson. Dies hat sich als Vorteil herausgestellt, da die Projektgruppe schnell und ausgerichtet an den konkreten Problemen, denen sie gegenüberstand, reagieren und agieren konnte. *Wir empfehlen daher keine vollständige Vorstrukturierung.*

### Betreuung

Die Betreuung war eher coachend statt instruierend. Auch hier wurde der Projektgruppe viel Raum für eigenes Handeln geboten. Statt strikter Vorgaben wurden der Gruppe eher Ideen für mögliche Lösungsansätze gegeben. Die Entscheidung für ein konkretes Vorgehen lag bei der Gruppe. *Wir würden künftig wieder diese Art der Betreuung wählen – coaching-orientiert.*

### Projektorganisation

Die Projektorganisation erfolgt komplett durch die Gruppe selbst. Dadurch konnten Aufgaben so verteilt werden, dass stets die geeignetste Person der Gruppe die Verantwortung übernahm. Die Gruppe konnte so eine Verteilung ausgerichtet an Erfahrung und Kompetenz vornehmen. Außerdem hat sich die Gruppe flexibel umorganisiert, wobei sie sich an der Art und Dringlichkeit der Probleme, sowie den verfügbaren Ressourcen orientierte.* Wir empfehlen ein hohes Maß an Selbstorganisation durch die Gruppe zu ermöglichen.*

### Kommunikation

Die Gruppe hat sich selbst über mehrere WhatsApp-Gruppen organisiert. Dabei gab es für jeden größeren Aufgabenbereich eine Chat-Gruppe, in der das jeweilige Team für diese Aufgabe vertreten war. Für übergreifende Kommunikation wurde auf vermittelnde Rollen, u. a. Product Owner, Scrum Master, gesetzt, sodass jedes Unterteam möglichst effektiv arbeiten konnte. Inhärent schwierig ist die soziale Komponente hierbei, d. h. die Teilnehmenden hätten sich gewünscht sich zumindest einmal im gesamten Team zusammenzukommen an einem Ort zur gleichen Zeit. *Wir empfehlen daher, dass Betreuende nicht nur reine Arbeit im Projekt forcieren, sondern auch gezielt Räume für sozialen Austausch schaffen.*

### Koordination

Die Gruppe hat sich selbst im Laufe des Projekts dazu entschieden die Kollaborationssoftware JIRA zu nutzen. Laut eigener Aussage konnte das Projekt dadurch viel effektiver und effizienter ablaufen, da alle Beteiligten so einen aktuellen Überblick über Fortschritt und Probleme hatten. Die Idee zu dieser Entscheidung kam von einer Teilnehmerin, die dieses Werkzeug aus dem beruflichen Kontext kennt. *Wir empfehlen daher, dass Vorschläge gemacht werden zur Koordination und möglichen Hilfsmitteln, aber keine zwingenden Vorgaben.*

### Kooperation

Für die Kooperation wurden keine besonderen Werkzeuge genutzt, die nicht auch in der Lehre Eingang fanden. Die Studierenden haben hier eher auf bewährte Werkzeuge und Techniken gesetzt und stets das Gesamtprojektziel im Auge behalten. *Es gab keine größeren Konflikte. Wir empfehlen Betreuenden nur in Ausnahmefällen einzugreifen und ansonsten die Gruppe selbst entscheiden zu lassen, auf welche Art sie kooperieren möchte.*

### Technische Unterstützung

Die Projektgruppe hat sich außer bei stark technisch-orientierten Aufgaben kaum an die Betreuungsperson gewendet. Die meisten Aktivitäten wurden eigenverantwortlich verwaltet und eigenständig technisch unterstützt, wo dies als notwendig erachtet wurde. *Wir empfehlen unterstützend und nach Maß vorzugehen und aktiv nachzufragen, aber keine harten Vorgaben zu machen, außer es handelt sich um komplexe Themen, die mehrere Dimensionen schneiden (Datenschutz, Sicherheit, Haftung etc.).*

## Zusammenfassung und Ausblick

Der vorliegende Beitrag beschreibt das Format des IT-Studienprojekts, seinen allgemeinen Aufbau, sowie die konkrete Ausgestaltung für eine rein virtuelle Durchführung. Dabei wird über die Erfahrungen einer virtuellen Durchführung im Rahmen eines Entwicklungsprojekts berichtet, bei dem ein Conversational User Interface im Kontext von Design Thinking Gegenstand war. Dabei wurde insbesondere die virtuelle Kollaboration beleuchtet, d. h. Kommunikation, Koordination und Kooperation. Auf Basis dieser Erfahrungen wurde eine Reihe von Handlungsempfehlungen abgeleitet, die Betreuenden künftiger virtueller IT-Studienprojekte bei der konkreten Gestaltung ihrer Veranstaltung unterstützen sollen.

Für weitere IT-Studienprojekte planen wir die Erprobung hybrider Modelle, sofern Präsenzanteile wieder möglich werden. Für rein virtuelle Durchführungen sehen wir die explorative Nutzung von virtuellen Räumen bzw. virtuellen Welten vor, um Aspekte der Nähe und des Sozialen stärker zu ermöglichen.

## Footnotes

1 www.miro.com.
